# The Development and Decay of the Circadian Clock in *Drosophila melanogaster*

**DOI:** 10.3390/clockssleep1040037

**Published:** 2019-11-19

**Authors:** Jia Zhao, Guy Warman, James Cheeseman

**Affiliations:** Department of Anaesthesiology, School of Medicine, Faculty of Medical and Health Sciences, The University of Auckland, Auckland 1142, New Zealand; jia.zhao@auckland.ac.nz (J.Z.); g.warman@auckland.ac.nz (G.W.)

**Keywords:** aging, central and peripheral clocks, clock gene reporter

## Abstract

The way in which the circadian clock mechanism develops and decays throughout life is interesting for a number of reasons and may give us insight into the process of aging itself. The *Drosophila* model has been proven invaluable for the study of the circadian clock and development and aging. Here we review the evidence for how the *Drosophila* clock develops and changes throughout life, and present a new conceptual model based on the results of our recent work. Firefly luciferase lines faithfully report the output of known clock genes at the central clock level in the brain and peripherally throughout the whole body. Our results show that the clock is functioning in embryogenesis far earlier than previously thought. This central clock in the fly remains robust throughout the life of the animal and only degrades immediately prior to death. However, at the peripheral (non-central oscillator level) the clock shows weakened output as the animal ages, suggesting the possibility of the breakdown in the cohesion of the circadian network.

## 1. Introduction

It is becoming increasingly clear that the circadian clock is not constant throughout life but rather is a dynamic system developing early in life, maturing, and finally decaying before death. The development of the clock is not instantaneous but rather structured and somewhat dependent on the formation of brain structures. At the other end of life, we know that, in humans and mammals at least, a compromised clock is detrimental to health and that aging is associated with a decline in sleep, decreased amplitude, and increased fragmentation [1]. However, the mechanisms controlling these processes are less clear. The advantages of the *Drosophila* model for circadian research need no introduction, particularly for the chronobiology community [2], and flies are also ideally suited to aging studies because of their relatively short life span and well defined life stages. Here we review the evidence for how the *Drosophila* clock develops and changes throughout life, and present a new conceptual model of our current understanding of the clock in *Drosophila*.

## 2. The Development of the Circadian Clock in *Drosophila*

An important question in chronobiology is determining the timing of when the central molecular clock starts to oscillate and when the peripheral clocks are synchronized throughout the whole animal. Behavioral rhythms in locomotor activity have been well described in adult *Drosophila*, and it has been proposed that behavioral rhythms may exist in pre-adult developmental stages. For example, a photophobic response mediated by clock neurons has been described in the larvae [3], although the light-avoidance behavior itself does not follow a circadian pattern. Despite the fact that eclosion only occurs once in a single fly’s lifetime, the multiple eclosion events within a population follow a circadian pattern [4]. Environmental factors administered during pre-adult stages can alter the adult circadian behavior and molecular state, even after the treatment is terminated. For instance, a brief light pulse or light-dark (LD) cycles can synchronize eclosion rhythms as well as locomotor activity rhythms in adults when provided as early as the first-instar larval stage (but not during embryonic stage), even though the flies are otherwise kept in constant darkness (DD) [5,6]. Subsequently, it has been demonstrated that a 10-minute light pulse or different light regimen in larvae can affect the adult free-running period or shift the phase of adult rhythms [7,8,9]. Aside from light, other stimuli, such as ethanol exposure during larval development, cause sustainable shortening of the adult free-running period [10,11]. The results from the above studies of pre-entrained adult behavioral rhythms imply that the circadian clock is present from the first larval stage, but whether it is already functional at earlier development is unclear.

*Drosophila* larvae possess the ability to phase shift the circadian clock in the pacemaker neurons with a light pulse, which can thus entrain eclosion rhythms, developmental hormones, and adult behavior [12]. Presumably, the larvae should be equipped with an efficient input pathway that can transmit environmental signals to the clock. Such an input pathway is required, not only for the entrainment of the circadian clock, but also for rapid initiation of behaviors (such as the photophobic response). Evidently, the larval visual system is capable of both functions [13], and the larval pacemaker neurons also show corresponding capability. Additionally, the sensory and central brain neurons necessary for pre-adult visual reactions, and the specific light inputs for each subgroup of clock neurons, have been identified in *Drosophila* larvae [14].

The visual system is part of the input pathways for light signals entering into the circadian clock of the brain. The larval optic nerve (Bolwig’s nerve), which projects to larval clock neurons, is thought to receive photic input from the larval eyes [15]. The contact between larval Bolwig’s nerve and clock neurons exists as early as the embryonic stage [16]. Moreover, aside from the larval visual organs, *cryptochrome* (*CRY*) is believed to crucially participate in larval circadian photoreception, making the entrainment of the circadian clock possible [8,16]. As in *CRY*-negative dorsal neurons, the larval light input pathway has been reported to be mediated by pigment-dispersing factor (PDF) neuropeptide signaling [17]. During the pupal stage, visual fibers that come from the Hofbauer-Buchner eyelet, a putative photoreceptive organ of the adult circadian clock, are found to be already contacting the pacemaker neurons [18]. A pair of giant visual projection neurons that have special properties in light sensitivity and rhythmic activities are also found in the pupae [19]. These data raise the possibility that the establishment of the input pathway to the central clock possibly starts in the embryonic stage, then goes through rapid development during the larval and pupal stages.

Existing evidence on the output and input pathways supports the presence of the circadian clock during early development. This includes the anatomical basis of pacemaker neurons and, importantly, their cyclic expression of core clock genes. In 1997, circadian pacemaker neurons were identified in a 4-hour-old first-instar larvae, along with their roles in controlling rhythmic processes in larvae, pupae, and adult [20]. Combined with findings of later studies, this shows that in the relatively simple larval brain, there are nine clock neurons per hemisphere, including five lateral neurons (LN), four of which express the PDF neuropeptide, and two pairs of dorsal neurons (DN_1_ and DN_2_) [14]. Among these neurons, the LN are the only cells that maintain rhythms in clock gene expression from first-instar larvae onward, then throughout metamorphosis and into adulthood [21]. Further, the PDF-expressing LN are implicated to be the master pacemaker neurons and subsequently represent the small ventral lateral neurons (s-LN_v_) of the adult brain [22,23,24]. The remaining groups of main pacemaker neurons necessary for the circadian mechanism, such as the large ventral lateral neurons (l-LN_v_) and dorsal lateral neurons (LN_d_), only differentiate during the mid-pupae stage [20,23,25]. This finding implies that a mature circadian clock composed of the whole package of pacemaker neurons may not exist before eclosion. Furthermore, despite similarities between the adult and larval molecular oscillators, as well as the neuronal projections [26], adult and larvae may differ substantially in the complexity and organization of their pacemaker neuronal circuits [27].

At the core of the circadian clock is the molecular oscillator, which is based on the transcription-translation feedback loops of several clock genes, including *period* (*per*) and *timeless* (*tim*). Work from Kaneko in 1997 has demonstrated that cyclic expression of period (PER) and timeless (TIM) occurs in the *Drosophila* larval central nervous system (CNS) in several groups of neurons (s-LN_v_, DN_1a_, and DN_2_), both in LD cycles and in subsequent DD conditions [21]. The technique of immunohistochemistry used in this study required animals to be sacrificed every six hours and data at the same time point to be pooled from different brain samples. The question remains whether this technique has sufficient resolution to show circadian oscillations adequately.

Even before larval development, significant expression of *per* mRNA exists in mid-late developing embryos (after 11-hour of embryogenesis). This early expression of *per* was found to be the CNS and localized within the brain and ventral ganglia [28]. Expression of *tim* was also detected in the embryonic CNS. The number, location, origin, and lineage of *per* and *tim* expressing cells were defined throughout embryogenesis in a recent study [29]. Clock (CLK) was shown to be initially expressed in presumptive s-LN_v_, DN_1_, and DN_2_ at the embryonic stage, and a few hours after the occurrence of CLK, PER accumulated in all CLK-expressing cells except presumptive DN_2_ during late embryos [30,31].

**Figure 1:**
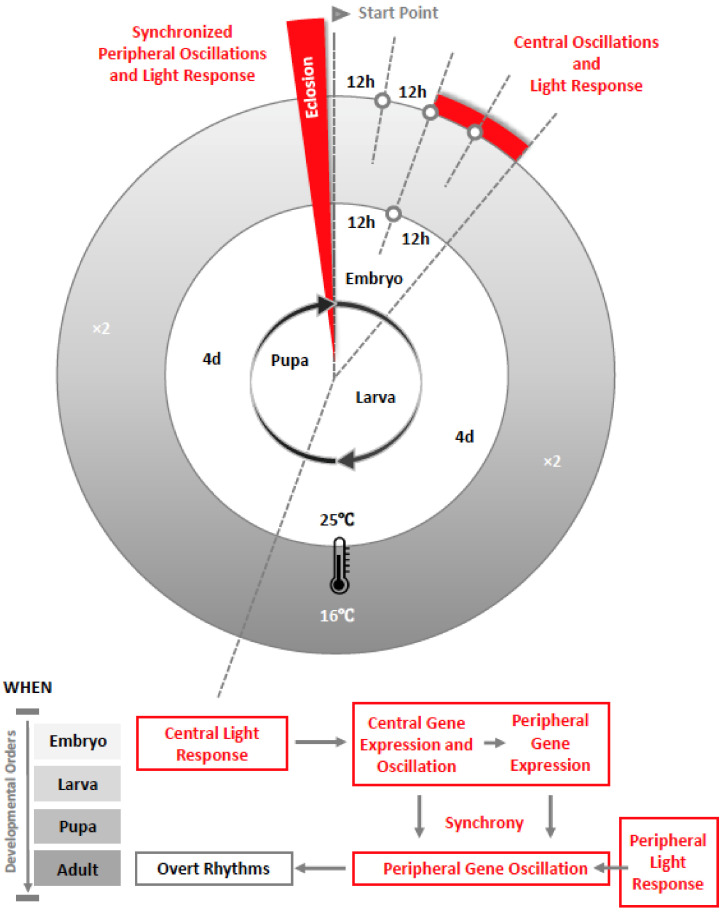
Life stages of *Drosophila* and recent findings on clock development from the embryo to eclosion as an adult. The diagram shows the negative correlation between temperature and development as defined in [34]. Higher temperature speeds up development and lower temperature slows it down within a range of 16 °C to 29 °C [35]. At 16 °C, the embryonic stage is prolonged by approximately twice the developmental time compared to 25 °C [36]. Based on our findings from a development study in *Drosophila*, circadian clock gene expression in the central clock neurons and their light-sensitivity start in the embryonic stage, which is earlier than previously thought. Clock gene expression in the peripheral clocks begin in embryos but only oscillate in synchrony in adults, when light-sensitivity is present [32]. The synchronized peripheral oscillations and light response at the whole body level in *Drosophila* occur after eclosion. We infer that *period* is expressed within different peripheral clocks around the body, which could be cyclic [37] or non-cyclic, but not synchronized at the whole animal level before eclosion. Our results also suggest that photoreceptors in peripheral clocks have not fully developed before the adult stage because PER expression is not synchronized in light-dark (LD) cycles. This is supported by a recent finding that, although expression of *cryptochrome* exists in certain larval peripheral tissue but not others, it lacks the function that mediates light-entrainment in both central and peripheral clocks in adult *Drosophila* [38].

Expression of core clock genes and their protein products found in the embryonic CNS of *Drosophila* suggests that brain oscillator neurons may begin their development during embryogenesis. This led us to question when exactly circadian PER expression occurs. We have found, using real-time luciferase reporters, that this in fact occurs as early as the embryonic stage in DD [32], which is earlier than previously thought (See Figure 1). Studies on peripheral clock development have attracted less attention. It was revealed that a significant non-cyclic expression of *per* mRNA initiates in mid-late developing embryos, which is limited to the midline of the nervous system [28,29,30,33]. This is consistent with our recent finding that PER expression begins in the embryo but does not oscillate in either DD or LD at the whole animal level until the adult stage [32].

## 3. The Aging of the Circadian Clock in *Drosophila*

Aging is the intrinsic, inevitable, and currently irreversible process of functional deterioration that consequently leads to the loss of viability [39]. The circadian clock is affected by time, showing progressive disturbance with advancing age [40,41]. The effect of aging on the circadian clock is believed to be highly conserved among humans and other animals [42]. It has been widely observed that the decline of circadian output rhythms is a prominent signature of aging. Prolonged free-running period, decline in sleep consolidation, and the overall rest-activity rhythm strength have been revealed in aged *Drosophila* [43,44].

Aside from downstream overt rhythms, there is also evidence for age-related changes in circadian organization at various levels, including alterations in the function of the molecular oscillators, the neuroanatomical structures, and the transmission and responsiveness to light [45]. How the clock changes at a molecular level during aging is less well known [46,47]. Whether aging is associated with defects in the central clock, degradation of the peripheral clocks, or loss of effective synchronization, is still unresolved.

First, it is unclear to what extent the central oscillator is affected by aging in general. Causal links between clock gene expression patterns at the central level and age-associated disturbance in circadian rhythms have not been conclusively demonstrated. For instance, it has been reported that PER and TIM oscillations in almost all central clock neuron groups (s-LN_v_, l-LN_v_, DN_1_, DN_2_, and LN_d_) deteriorate with advancing age [44]. However, this is challenged by the finding that PER is robustly expressed in synchrony among different groups of clock neurons (s-LN_v_, DN_1_ and LN_d_ [48], and DN_1-3_ and LN_d_ [49]) in aged flies.

Secondly, concerning the peripheral clocks in *Drosophila*, many studies indicate age-related molecular decline. Reduction in amplitude of circadian clock gene expression has been found throughout the body in aged animals [50]. Likewise, *per* and *tim* luciferase reporters have shown a decline in expression of peripheral clocks throughout the fly body with advancing age [48,49]. Further, *per* mRNA oscillations [51], as well as *per* and *tim* at both mRNA and protein levels [48,52], have been reported to decrease in the heads of old flies. It has also been shown in a recent study by using both techniques of RNA sequencing and qPCR that *tim* expression declines in aged flies’ heads [53].

Even though many results in *Drosophila* studies show molecular decline at the gross peripheral level, different tissues or body parts seem to respond differently. For example, transcriptional oscillations of *per* and *tim* have been shown to decrease in heads but not bodies of aged flies [46]. As one of the peripheral clocks, the retinal photoreceptor cells in the compound eyes of old flies show decline in expression of PER, yet that is not the case for another peripheral oscillator, the Malpighian tubules, as strong PER oscillations have been observed in aged animals [47].

In brief, it is known that tissues/organs age at different rates and have tissue-specific signatures, adding to the complexity of age-related properties within individual oscillators. Besides, various peripheral tissues/organs may have different functional implications to the entire circadian system. Therefore, there is no clear consensus summarizing the comprehensive changes of the peripheral clocks due to aging at this stage.

Death is the cessation of biological functions that maintain homeostasis and sustain a living organism [54]. The death of an animal means that its systems, organs, tissues, and cells stop functioning as a whole, which implies that the individual parts may still be alive at the time when the body dies [54].

It is highly likely that the circadian clock is one of the determinants of longevity [55]. First, a better functioning clock itself is associated with longer lifespan. Flies displaying rhythmic locomotor activity live longer than arrhythmic ones [56]. Secondly, the alignment between the intrinsic free-running period and the environmental cycles is a well-accepted predictor of lifespan. Organisms kept in an environment that best matches their internal circadian clock outcompete the others in longevity studies of *Drosophila* [57,58]. The connection between the circadian clock and death anecdotally is widely known, and has been shown at least in a few studies, with clock-controlled behavior changing before death, and aged flies in their last days of life were found to exhibit arrhythmicity in behavior [48].

With general acceptance that decline of the circadian clock is a reliable manifestation of aging, it is not hard to imagine that collapse of the clock could be a potential indicator of death. Nevertheless, there has been very little investigation into the mechanism of this phenomenon prior to death.

Presumably, different levels of the clock organization may be involved in the mechanism of death. Aside from the central clock and the overt rhythms it controls, another promising candidate is the molecular clock and its property genes at the peripheral level. Interestingly enough, system-wide knockouts of clock genes create so-called “accelerated aging” phenotypes [55]. For instance, null mutation of the *per* gene [51] or *tim* gene [59] or the *cycle* gene [60] in *Drosophila* is associated with significantly shorter lifespan, as well as impaired locomotor rhythms. An intriguing idea also is that changes in clock gene expression may be implicated, not only in age-associated changes in circadian rhythmicity, but also more broadly in the processes of aging overall [55]. It has been revealed that a novel marker of imminent death is the dramatic change in expression of the clock gene *tim* in *Drosophila*. Importantly, this marker in the expression of *tim* was not age dependent and predicted death equally well in fruit flies of different ages and under light and temperature cycles. It is considered the central mechanism of the disturbance in circadian behavior before death [49].

## 4. Discussion

A considerable amount of research has helped shed light on the circadian clock being crucial for maintaining organized and healthy life history. The circadian clock has been implicated in affecting life traits such as pre-adult development as well as adult lifespan. It is generally believed that a faster clock (shorter free-running period) speeds up pre-adult development and shortens lifespan, vice versa, a slower clock (longer free-running period) slows down development and lengthens lifespan [61,62,63,64].

The developmental plan of an animal body is built through the coordinated timing of cell divisions, cell differentiation, and cell movements [65], and the circadian clock is one of the important determinants that orchestrate these basic processes. It has been reported that the pace of pre-adult development in *Drosophila* is controlled by clock genes [66]. For instance, *per* mutant studies suggest a positive correlation between free-running period and development time in *Drosophila*. The *per^Short^* mutants (19-hour) develop faster from eggs to adult than the wild type, while *per^Long^* mutants (28-hour) complete development more slowly than the wild type [61]. Furthermore, the circadian clock is involved in regulating the timing of eclosion in *Drosophila* [67]. This key developmental event is restricted to occur around dawn, even if flies are ready to eclose earlier. Acting by means of PDF, interactions between a peripheral clock in the prothoracic gland and the central clock of the lateral neurons, seems to explain the mechanism of the circadian timing of eclosion [68,69,70].

Recently, we found that the development of the circadian clock itself goes through a hierarchical process, with certain central clock neurons developing first, and peripheral tissues at the body level maturing later [32]. The meaning is presumably simple; that the central clock plays a relatively more important role in regulating the early development of the clock system itself, so much as to coordinate the development of the entire body. The underlying mechanism is still unknown, but the development of tight or loose coupling may explain why expression of clock genes shows circadian rhythmicity within central clock neurons, while it only increases but does not oscillate in synchrony among peripheral clocks at the same developmental stages. We infer that network assembly at the systemic level is on-going but incomplete during early development. The non-cyclic expression of *per* in peripheral tissues under constant darkness implies the scarce input from the central pacemaker. Without internal cues, external stimuli, such as light-dark (LD) cycles’ being incapable to entrain clock gene oscillations, indicates that the peripheral property of light-sensitivity has not yet been established before eclosion.

Aging is characterized as a gradual decline in numerous systems, influenced by both genetic and environmental factors, with changes happening at both the physiological and molecular levels [41]. It is still not clear whether decline of clock function is the cause or the result of the aging process, or both. Existing evidence suggests a bidirectional relationship between the circadian clock and aging. While there is evidence of age-related dampening of clock oscillations and clock-controlled rhythms, disruption of the circadian clock in turn leads to accelerated aging and increased susceptibility to age-associated pathologies [47,52,55]. When aging and the aged clock interact adversely with each other, it leads to an irreversible outcome of deterioration.

The effect of aging on the circadian clock is complex and warrants broader and deeper exploration. Aging may act on every component of the clock organization, including the center and the periphery, at the levels of tissue, intercellular connection, and the cell. Although, presumably, no party can escape the influence of aging, the weight of contribution can be diverse. From the existing results of *Drosophila* studies, a general impression is that the molecular machinery in the central clock is less or later damaged, if at all, by the aging process. We find that the expression of clock genes in the central clock neurons of aged *Drosophila* stays as robust as in young ones [49]. As a consequence, causal links between clock gene expression patterns in the central clock and age-associated disturbance in circadian rhythms cannot be conclusively demonstrated.

Nevertheless, it does not mean that the central clock is impervious to the challenge of aging. *Drosophila* show convincing patterns of decline in synchronization among central clock neurons. The evidence on reduction of the PDF levels in the central clock network is consistent [44,48]. Moreover, overexpression of PDF in PDF-positive neurons increases clock gene expression in specific central clock neurons, and partially rescues rhythmicity and period changes of output behavior in old flies [44]. These findings indicate the important role of PDF in the aging process, as well as its implication in age-associated attenuation of intercellular communication in the circadian neuronal network. The theory is that, with advancing age, it becomes increasingly difficult for the rhythms of individual neurons to remain synchronized with each other and thus the rhythmic output of the network becomes fragmented [41].

Aside from the impairment of the central clock, another possible mechanism underlying circadian disruption with increasing age is the degradation of the peripheral clocks. It is generally believed that peripheral clocks go through more serious damage than the central pacemaker, as we show in the aging study [49]. Despite the reasons having not been fully revealed, one of the possibilities is that peripheral tissues lack the tight-coupling network system that is unique to the central clock. It is not hard to imagine that the breakdown of the network should not only exist within the central, but, inevitably and more dramatically, can occur in the peripheral clocks, since they are far more complicatedly structured yet far less efficiently coordinated. As a consequence, such a loose network structure puts peripheral clocks in a more vulnerable status, making them easily targeted by pathological processes such as aging.

It is known that organs age at different rates with tissue-specific signatures [71], adding the complexity of age-related properties within individual oscillatory tissues. Further, the changes in peripheral oscillator behavior may not only result from intrinsic alterations within the tissues themselves, but could also be a consequence of the reduction of their responsiveness to internal or external temporal cues. Our result shows the inefficiency of strong light and temperature entrainments on rescuing the age-related deterioration of the peripheral oscillators [49]. This indicates that the impairment of the peripheral input pathway may be a possible mechanism involved in the aging process. It is supported by the finding that the photoreceptor CRY reduces at both mRNA and protein levels in peripheral clocks of aged *Drosophila*. Further, restoration of an effective input signal through overexpressing CRY in the peripheral clocks can reverse the decline of circadian rhythms and benefit health [52].

Finally, we find that the clock stops ticking properly, not at the time of death, but before death [49]. The molecular clockwork at the periphery throughout the body increases the clock gene expression in the last few days before death, opposite to its declining trend with advancing age. This may reflect a stress response or a compensatory mechanism as yet unknown. The total loss of behavioral rhythms at the final stage of life implies a deeply dysfunctional clock. The question remains whether the timing of death is also clock regulated.

## 5. Conclusions: The Dynamic Network

**Figure 2:**
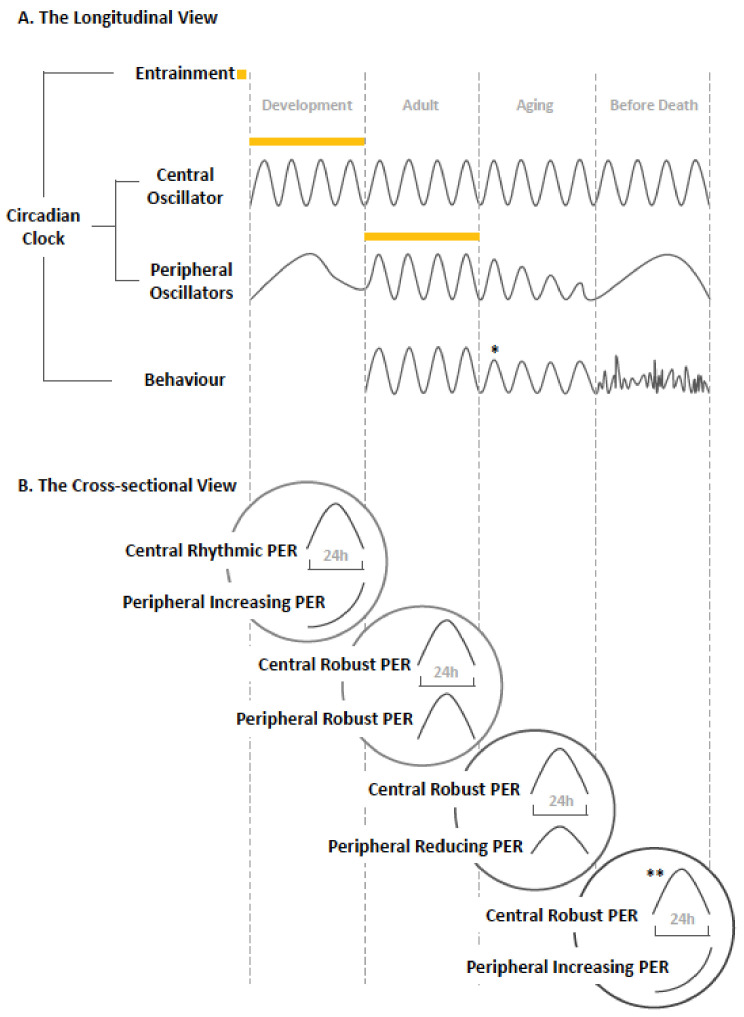
Conceptual model of the development and changes in the clock over the lifetime of *Drosophila*. (**A**) Summary in longitudinal format and (**B**) specific changes characterizing the function of the clock at each life stage [43,48].

The two dimensions of the circadian clock, spatially from the center to the periphery (each comprising the input, the molecular oscillator, and the output), as well as temporally from eclosion to death, have been reviewed in this study. We emphasize the dynamic changes of the circadian network as well as each interconnected component (See Figure 2). At the longitudinal view throughout lifespan in *Drosophila*, the central molecular clock develops early at the embryonic stage and continues to stay robust when the animal ages. By contrast, the peripheral molecular clock matures later at the adult stage and shows decline with advancing age, ending up with an outbreak in the last days of life. Entrainment of the central clock establishes during embryogenesis, which is earlier than that of the peripheral clock, which occurs in the adult stage. Rhythms of locomotor activity emerge from adult onwards, deteriorate with increasing age [43], and lose rhythmicity completely preceding death.

At the cross-sectional view of specific age in *Drosophila*, early development stage shows a rhythmic central clock and an emerging but not yet synchronized peripheral clock system. The mature clock system, composed of the well-functional central and peripheral components, is evident in the adult. The aging stage shows a robust central clock and declined peripheral clocks, and finally at the stage before death, severe disruption is found at the peripheral level but not at the central level [48].

Within the network system of the circadian clock, peripheral oscillators are one of the crucial elements. As an important player within the circadian network, peripheral clocks should be valued more in their normal function and their malfunction during certain pathological conditions.

The formation, maturity, decline, and breakdown of the circadian network at different life stages indicate the importance of an integrated system to health. The understanding of the life traits of the circadian clock horizontally and vertically should have implication on development- or age-related diseases in the future.
